# Real-World Outcomes and Factors Associated With the Second-Line Treatment of Patients With Gastric, Gastroesophageal Junction, or Esophageal Adenocarcinoma

**DOI:** 10.1177/1073274819847642

**Published:** 2019-05-06

**Authors:** Afsaneh Barzi, Lisa M. Hess, Yajun E. Zhu, Astra M. Liepa, Tomoko Sugihara, Julie Beyrer, Joseph Chao

**Affiliations:** 1 Norris Comprehensive Cancer Center, University of Southern California, Los Angles, CA, USA; 2 Global Patient Outcomes and Real-World Evidence, Eli Lilly and Company, Indianapolis, IN, USA; 3 Clinical Solutions, Syneos Health, Raleigh, NC, USA; 4 City of Hope Comprehensive Cancer Center, Duarte, CA, USA

**Keywords:** chemotherapy, decision-making, patient care, survival, health services research, gastroesophageal adenocarcinoma

## Abstract

This retrospective observational study was designed to evaluate overall survival in a real-world patient population and to identify predictive factors associated with receipt of second-line therapy. A retrospective analysis of electronic medical records (Flatiron Health, New York) was conducted among patients initiating first-line therapy from January 1, 2013, through April 30, 2018. Eligible patients were diagnosed with advanced gastric, gastroesophageal junction, or esophageal adenocarcinoma and ≥18 years of age at the time of treatment initiation. Patients alive 45 days after discontinuation of first-line therapy were considered potentially eligible for continued therapy and were categorized into those who received and those who did not receive second-line therapy. Survival analyses were conducted using Kaplan-Meier method and log-rank test without adjusting for any baseline covariates. Factors associated with further treatment were evaluated using logistic regression. A total of 3850 patients met eligibility criteria. Among the 2516 patients available to receive second-line therapy, 1515 (60.2%) received second-line therapy and 1001 (39.8%) did not receive further therapy. Among those potentially eligible to receive second-line therapy, median survival was 15.4 months (95% confidence interval [CI]: 14.6-16.0) from initiation of first-line therapy for those who received second-line therapy and 10.0 months (95% CI: 9.3-10.7) for those who did not. Longer duration of first-line therapy (≥169 vs ≤84 days), HER2-positive tumors, initially diagnosed with stage IV disease, less weight loss during first-line therapy, and younger age were associated with receipt of second-line therapy (all *P* < .001). Longer survival was associated with multiple lines of therapy; however, these results should be interpreted with caution, and no causal relationship can be inferred.

## Introduction

Gastric, gastroesophageal junction (GEJ), and esophageal cancers carry a very poor prognosis, particularly when diagnosed at stage IV (ie, metastatic disease). The 5-year relative survival rate for patients diagnosed with gastric/GEJ or esophageal cancers is 31.5% and 20.7%, respectively.^1^ A significant proportion of patients are diagnosed with advanced-stage disease, and among those who undergo surgical resection, recurrence remains a significant issue. Unfortunately for patients diagnosed with metastatic disease, potentially curative surgical resection is not an option.^2^ National Comprehensive Cancer Network (NCCN) category 1 evidence supports first- and second-line systemic therapy for patients with unresectable locally advanced, metastatic, or recurrent disease.^3,4^ In the first-line setting, the NCCN guidelines recommend 2-drug cytotoxic therapy. Two gastric cancer regimens are preferred by the NCCN: fluoropyrimidine plus cisplatin (category 1) or fluoropyrimidine plus oxaliplatin (FOLFOX, category 2A).^4^ Modified dosing of the DCF (docetaxel, cisplatin, and a fluoropyrimidine) regimen or substitution of cisplatin with oxaliplatin are considered category 2A evidence for GEJ and esophageal cancers (squamous and adenocarcinoma).^3^ Additionally, trastuzumab is recommended to be added to first-line chemotherapy for patients whose tumors overexpress HER2. In the second line, 5 preferred regimens are supported by category 1 evidence for gastric, GEJ, or esophageal adenocarcinoma: ramucirumab plus paclitaxel or single-agent ramucirumab, docetaxel, paclitaxel, or irinotecan.^3,4^ Pembrolizumab is supported by category 2A evidence for the second-line or subsequent treatment of tumors with high-microsatellite instability (MSI-H) or for deficient mismatch repair tumors and in the third-line or later setting for PD-L1–positive adenocarcinoma.^4^

The survival benefit of second-line therapy is supported by numerous randomized clinical trials.^5-9^ In the REGARD trial, ramucirumab monotherapy reduced all-cause mortality by 22% compared to placebo plus best supportive care (BSC; hazard ratio [HR] for overall survival [OS] = 0.78; 95% confidence interval [CI]: 0.603-0.998; *P* = .0473) and reduced the risk of disease progression or death by 52% (HR for progression-free survival [PFS] = 0.48; 95% CI: 0.376-0.620; *P* < .001) in patients with gastric or GEJ adenocarcinoma.^5^ An open-label, randomized study of irinotecan versus BSC in patients with gastric or GEJ adenocarcinoma found that irinotecan improved OS (HR = 0.48; 95% CI: 0.25-0.92, *P* = .012); PFS was not compared.^7^ COUGAR-02 was an open-label, randomized trial comparing docetaxel to active symptom control in patients with gastric, GEJ, or esophageal adenocarcinoma. This study also demonstrated OS improvement associated with docetaxel (HR = 0.67; 95% CI: 0.49-0.92; *P* = .01); PFS was not compared between the groups.^8^ A Cochrane review of 11 randomized trials including 1347 participants found a significant improvement in OS chemotherapy and/or targeted therapy versus BSC or control in the second-line treatment of patients with esophageal or GEJ cancer (HR for OS = 0.75; 95% CI: 0.68-0.84). Five trials (883 participants) provided data supporting the benefit in PFS for second-line treatment as well (HR for PFS = 0.64; 95% CI: 0.45-0.92).^9^ Despite the mounting evidence for benefit of second-line therapy in clinical trials, such evidence for a real-world population is lacking.

Retrospective observational studies report that less than 50% of patients receive second-line treatment for advanced or metastatic gastric cancer.^10-12^ The reasons associated with treatment discontinuation at first-line therapy are unclear. Potential reasons could include comorbidities or declining health leading to inability to receive further treatment, patient death, or patient or physician choice. Identifying the factors associated with the receipt of second-line therapy in real-world population is a critical step in developing strategies to increase adherence of patients and providers to this recommended practice.

This retrospective observational study was designed to examine whether the clinical benefit of continued therapy would be demonstrated in an unselected, real-world population. Additionally, this study was designed to identify factors associated with receipt of second-line therapy to inform potential strategies to improve the rate of continued therapy among eligible patients with gastroesophageal adenocarcinoma.

## Methods

### Data Source

The Flatiron Health database is a longitudinal, demographically, and geographically diverse database derived from electronic medical record (EMR) data. The Flatiron Health database at the time of this study included data from over 255 cancer clinics representing 1.7 million patients with active cancer. The Flatiron Health Advanced Gastric/Esophageal Cohort is a subset of the overall Flatiron Health database that includes a geographically diverse random sample of over 7500 patients with advanced gastric/GEJ/esophageal cancer at Flatiron community oncology and academic cancer cancers in the United States (as of April 30, 2018, which was the last data available at the time of analysis). Patients in the database are diagnosed with stage IV disease or with distant recurrence, a second locoregional recurrence after any initial stage at diagnosis (gastric only), a first locoregional recurrence that was not completely resected (or any locoregional recurrence for patients with esophageal/GEJ cancer), or no surgical resection of the primary tumor. The database includes patients whose advanced cancer diagnoses occurred on or after January 1, 2011, and who have 2 or more visits documented in the EMR during that time period. The database includes both structured and unstructured EMR data elements curated via technology-enabled abstraction, such as patient demographics (gender, race/ethnicity, birth year, and state of residence), community versus academic facility, clinical diagnostic codes, laboratory data, HER2 expression testing and status, medications ordered and/or administered, line of therapy (derived), month and year of death, and clinical characteristics including cancer stage at diagnosis, tumor histology, and Eastern Cooperative Oncology Group performance status. This data set is deidentified, and provisions are in place to prevent reidentification in order to protect patients’ confidentiality. This noninterventional study does not qualify as human subjects research in accordance with the US Code of Federal Regulations (CFR), 45 CFR 46.102(f) and is thereby exempt from institutional review board (IRB) evaluation.

### Study Sample

Patient records eligible for inclusion were those in the Flatiron advanced gastric/esophageal cohort who had a primary diagnosis of gastric, GEJ, or esophageal adenocarcinoma. As described earlier, this database is limited to patients with advanced or metastatic cancers, data specific to care of early-stage disease or treatment in the adjuvant or neoadjuvant setting are not included. All patients in this study must have initiated first-line therapy on or after January 1, 2013, to ensure the study included a current cohort of patients. Flatiron oncologist-defined, rule-based lines of therapy were used to identify lines of therapy. In general, a change in a line of therapy is defined based on the addition of new chemotherapy agents and gap periods in which no treatment was received. The rules used to define lines of therapy in Flatiron are consistent with other published approaches.^13^ Patients diagnosed with squamous carcinomas or who were younger than the age of 18 years at the initiation of first-line therapy were also excluded. Radiation therapy is not recorded in the database; therefore, it was assumed that patients receiving weekly carboplatin + paclitaxel were likely receiving concurrent radiation therapy.

### Statistical Methods

This study was designed as a descriptive, noncomparative analysis. Descriptive statistics were reported using mean, standard deviation, median, and range for continuous variables and frequency counts and percentages for categorical variables. Patients were assumed to be eligible for second-line therapy if they were alive at least 45 days after completion of first-line therapy and had discontinued first-line treatment prior to April 30, 2018 (the date of last available record). The selection of 45 days was based on the optimal date that would exclude the sickest patients who were likely never candidates for second-line therapy while not creating bias by retaining patients unable to initiate 2L therapy. Patients were assumed to have discontinued first-line therapy if either they had initiated a subsequent line of therapy or if they had not received therapy within at least 30 days prior to the last data available in the database or end of follow-up. The end of first-line therapy was defined as the last administration date of first-line therapy. In the case of oral medications, 30 days were added to the last refill date. Unadjusted *P* values were calculated to evaluate differences in characteristics between patients who received second-line therapy and those who did not (among those eligible for second-line therapy) using *t* test/F test for continuous variables and χ^2^ test for categorical variables. Predictors of second-line treatment were evaluated using multivariable logistic regression. The regression model was built to predict the probability of patients receiving second-line treatment (vs no second-line treatment). The candidate covariates included age-group (18-64 vs 65+), gender (female vs male), race (Asian, black or African-American, white, other race, vs missing/unknown), practice type (community vs academic), disease site (esophageal, GEJ, vs gastric), HER2 status (positive, negative, vs missing/unknown), advanced diagnosis (stage IV at diagnosis vs recurrent/unresectable disease), body mass index (underweight, normal weight, overweight, obese, vs missing/unknown), prior resection (yes vs no), weight loss during first-line therapy (loss <10% of baseline body weight, loss ≥10% baseline body weight, no change, other weight gain, vs missing), duration from advanced diagnosis date to start of first-line therapy (<70 days vs 71-100 vs 100-200 vs >200 days), duration of first-line therapy (≤84 days vs 85-168 days vs ≥169 days), and creatinine level. Stepwise variable selection procedure was used to identify factors significantly associated with the outcome with entry significance level of 0.15 and stay significance level of 0.1.

To reduce the risk of immortal time bias, survival analyses between those who did and did not receive second-line therapy excluded any patient who was still receiving first-line therapy as of April 30, 2018 (last record available at the time of analysis) or who had died during or within 45 days of completing first-line therapy. Overall survival from the start of 1L therapy was estimated using Kaplan-Meier method and log-rank test without adjusting any baseline covariates. Additional analyses explored differences between patients who had died during the period from initiation to the date of last infusion of first-line therapy and those who did not die during this period.

Due to the lack of data regarding radiation therapy in the EMR database, sensitivity analyses were conducted to estimate the impact of potential receipt of radiation therapy. For patients receiving weekly carboplatin plus paclitaxel, it was assumed for the sensitivity analyses that these patients were receiving concurrent radiotherapy. The lines of therapy were recoded excluding those records where chemoradiation was a line of therapy. Therefore, a patient receiving chemoradiation would have been recategorized from first-line carboplatin plus paclitaxel to line zero chemoradiation. Survival analyses were repeated using the revised rules to determine the stability of results if possible miscategorization occurred in the absence of radiation therapy data.

## Results

**Figure 1.**
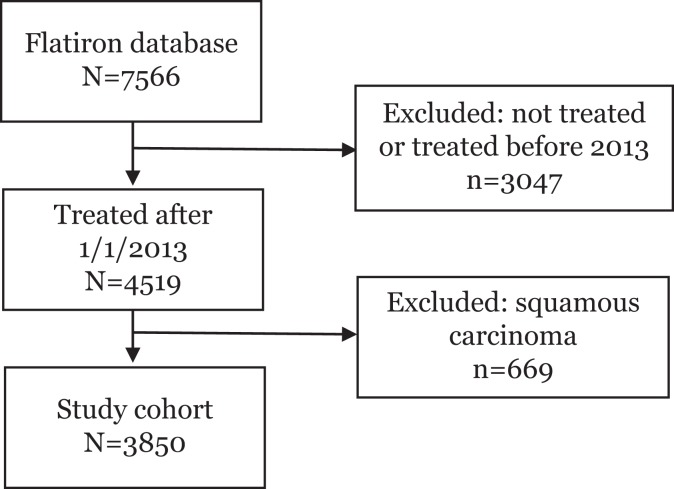
Eligibility flow diagram.

There were 7566 patients in the Flatiron database available for analysis. Of these, 3850 met eligibility criteria and were included in the study (Figure 1). The primary tumor location was gastric (n = 1388, 36.1%), GEJ (n = 1103, 28.6%), and esophagus (n = 1359, 35.3%). The baseline characteristics of the study cohort are summarized in Table 1. All patients received first-line therapy in this study; 41.1% (n = 1584) received at least 2 lines of therapy and 17.6% (n = 676) received three or more lines of therapy. Of the 2266 patients who did not receive second-line therapy, 19.9% (n = 451) died before or during first-line therapy, and 27.1% (n = 614) had less than 45 days of follow-up after first-line therapy. Among the 2516 patients eligible to receive second-line therapy (ie, alive at least 45 days after discontinuation of first-line therapy and not still receiving first-line therapy at the last available recorded data), 1515 (60.2%) received additional therapy and 1001 (39.8%) did not. The most common treatment regimens received for all patients are summarized in Table 2. A full summary of all regimens received by patients is provided in the Supplemental Appendix.

**Table 1.** Demographic and Clinical Characteristics of the Study Cohort.

			Tumor Site			
		Overall, N = 3850	Gastric, n = 1388	GEJ, n = 1103	Esophageal, n = 1359	*P* Value^a^
Age, years	Mean (SD)	66.23 (11.769)	64.86 (13.119)	66.93 (11.054)	67.06 (10.705)	<.0001
Gender, n (%)	Female	898 (23.32)	538 (38.76)	192 (17.41)	168 (12.36)	<.0001
	Male	2952 (76.68)	850 (61.24)	911 (82.59)	1191 (87.64)	
Race	Asian	128 (3.32)	109 (7.85)	12 (1.09)	7 (0.52)	<.0001
	Black/African-American	240 (6.23)	179 (12.90)	35 (3.17)	26 (1.91)	
	White	2596 (67.43)	681 (49.06)	843 (76.43)	1072 (78.88)	
	Other Race	452 (11.74)	240 (17.29)	88 (7.98)	106 (7.80)	
	Missing	434 (11.27)	166 (11.96)	122 (11.06)	146 (10.74)	
Ethnicity^b^, n (%)	Hispanic	317 (8.23)	231 (16.64)	28 (2.54)	58 (4.27)	<.0001
	Non-Hispanic	3533 (91.77)	1157 (83.36)	1075 (97.46)	1301 (95.73)	
Geographic region, n (%)	Northeast	805 (20.91)	267 (19.24)	253 (22.94)	285 (20.97)	<.0001
	South	1331 (34.57)	509 (36.67)	374 (33.91)	448 (32.97)	
	Midwest	661 (17.17)	167 (12.03)	192 (17.41)	302 (22.22)	
	West	699 (18.16)	293 (21.11)	179 (16.23)	227 (16.70)	
Practice setting, n (%)	Academic	270 (7.01)	96 (6.92)	93 (8.43)	81 (5.96)	.06
	Community	3580 (92.99)	1292 (93.08)	1010 (91.57)	1278 (94.04)	
Prior resection, n (%)	Yes	476 (12.36)	179 (12.90)	150 (13.60)	147 (10.82)	.09
	No	3374 (87.64)	1209 (87.10)	953 (86.40)	1212 (89.18)	
HER2 status, n (%)	Negative	1434 (37.25)	617 (44.45)	357 (32.37)	460 (33.85)	<.0001
	Positive	408 (10.60)	103 (7.42)	145 (13.15)	160 (11.77)	
	Unknown	488 (12.68)	147 (10.59)	149 (13.51)	192 (14.13)	
	Missing	1520 (39.48)	521 (37.54)	452 (40.98)	547 (40.25)	
BMI category, n (%)	Underweight	351 (9.12)	146 (10.52)	98 (8.88)	107 (7.87)	<.0001
	Normal	1185 (30.78)	509 (36.67)	305 (27.65)	371 (27.30)	
	Overweight	1087 (28.23)	352 (25.36)	309 (28.01)	426 (31.35)	
	Obese	926 (24.05)	265 (19.09)	294 (26.65)	367 (27.01)	
	Missing/unknown	272 (7.06)	98 (7.06)	92 (8.34)	82 (6.03)	
ECOG performance status, n (%)	0	125 (3.25)	60 (4.32)	30 (2.72)	35 (2.58)	.07
	1	146 (3.79)	63 (4.54)	41 (3.72)	42 (3.09)	
	2	29 (0.75)	12 (0.86)	7 (0.63)	10 (0.74)	
	3	5 (0.13)	3 (0.22)	1 (0.09)	1 (0.07)	
	Missing/unknown	3545 (92.08)	1250 (90.06)	1024 (92.84)	1271 (93.52)	
Total number of lines of therapy, n (%)	1	2266 (58.86)	858 (61.82)	601 (54.49)	807 (59.38)	.02
	2	908 (23.58)	313 (22.55)	270 (24.48)	325 (23.91)	
	3	441 (11.45)	137 (9.87)	152 (13.78)	152 (11.18)	
	4	148 (3.84)	48 (3.46)	48 (4.35)	52 (3.83)	
	5	61 (1.58)	20 (1.44)	25 (2.27)	16 (1.18)	
	6	20 (0.52)	11 (0.79)	5 (0.45)	4 (0.29)	
	7	5 (0.13)	1 (0.07)	2 (0.18)	2 (0.15)	
	8+	1 (0.03)	0 (0.00)	0 (0.00)	1 (0.07)	
Disease status at advanced diagnosis	Diagnosed metastatic	2038 (52.94)	853 (61.46)	535 (48.50)	650 (47.83)	.07
	Recurrent metastatic	408 (10.60)	153 (11.02)	129 (11.70)	126 (9.27)	

Abbreviation: GEJ, gastroesophageal junction; BMI, body mass index; ECOG, Eastern Cooperative Oncology Group; SD, standard deviation.

^a^Gastric versus GEJ versus esophageal.

^b^Ethnicity was dichotomized as Hispanic, if reported, versus all others who were considered non-Hispanic.

**Table 2.** Most Common Treatment Regimens Used for the Treatment of Gastroesophageal Adenocarcinoma (Limited to Regimens used in >4% of at Least 1 Group).

First line, n (%)			
	Gastric, n = 1388	GEJ, n = 1103	Esophageal, n = 1359
FOLFOX	376 (27.1)	215 (19.49)	264 (19.43)
Capecitabine	120 (8.65)	39 (3.54)	36 (2.65)
Capecitabine, Epirubicin, Oxaliplatin	74 (5.33)	33 (2.99)	34 (2.50)
Carboplatin, Paclitaxel, Radiation^a^	52 (3.75)	263 (23.84)	400 (29.43)
FOLFOX, trastuzumab	47 (3.39)	53 (4.81)	52 (3.83)
Carboplatin, paclitaxel	22 (1.59)	56 (5.08)	57 (4.19)
Second line, n (%)			
	Gastric, n = 530	GEJ, n = 502	Esophageal, n = 552
Paclitaxel, ramucirumab	107 (20.19)	84 (16.73)	76 (13.77)
FOLFOX	62 (11.70)	65 (12.95)	84 (15.22)
Ramucirumab	32 (6.04)	23 (4.58)	14 (2.54)
FOLFIRI	34 (6.42)	30 (5.98)	30 (5.43)
Capecitabine	24 (4.53)	24 (4.78)	30 (5.43)
Carboplatin, paclitaxel, radiation^a^	21 (3.96)	16 (3.19)	28 (5.07)
FOLFOX, trastuzumab	8 (1.51)	19 (3.78)	25 (4.53)
Third line, n (%)			
	Gastric, n = 217	GEJ, n = 232	Esophageal, n = 227
Paclitaxel, ramucirumab	37 (17.05)	43 (18.53)	36 (15.86)
Ramucirumab	23 (10.60)	11 (4.74)	12 (5.29)
FOLFIRI	20 (9.22)	21 (9.05)	19 (8.37)
Irinotecan	15 (6.91)	11 (4.74)	9 (3.96)
FOLFOX	14 (6.45)	12 (5.17)	19 (8.37)

Abbreviation: GEJ, gastroesophageal junction.

^a^Radiation therapy was assumed to be used in a weekly carboplatin plus paclitaxel dosing regimen; these regimens were recoded as line zero in sensitivity analyses.

**Figure 2.**
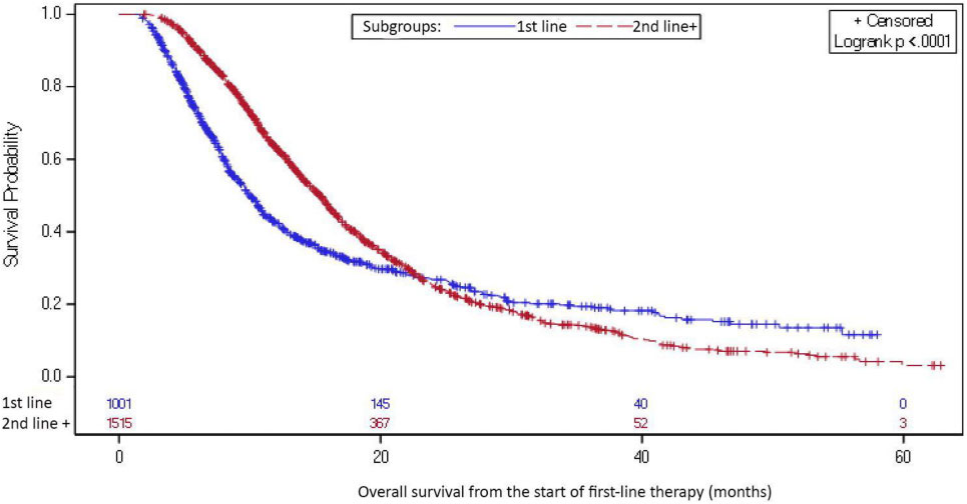
Overall survival, patients receiving first-line therapy only (n = 1001) versus those receiving more than one line of therapy (n = 1515)^a^.

The median OS from start of first-line therapy for patients who received second-line therapy was 15.4 months (95% CI: 14.6-16.0), and for those who did not receive second-line therapy, median OS was 10.0 months (95% CI: 9.3-10.7, Figure 2). Survival analyses in the sensitivity analysis, excluding chemoradiation were consistent (median OS for those who did and did not receive chemoradiation was 14.9 [95% CI: 14.0-15.7] and 8.6 months [95% CI: 8.1-9.3], respectively).

Patients who died during or shortly after first-line therapy were more likely to have experienced weight loss, dose reductions during first-line therapy, and had a shorter duration of first-line therapy than those who were alive (Table 3). In multivariable analysis, factors statistically significantly associated with receipt of second-line therapy included longer duration of first-line therapy, lack of body weight loss during first-line therapy, younger age, having a tumor that overexpressed HER2, and patients with an initial diagnosis of metastatic disease (Table 4). All other covariates in the model were not statistically significant.

**Table 3.** Selected Characteristics During First-Line Therapy: Patients Who Died and Those Who Did Not Die During First-Line Therapy.

		Died During First-Line Therapy, n = 451	Alive 45 Days After First-Line Therapy, n = 3399	*P* Value
Creatinine	n	394	2975	.28
	Mean (SD)	0.94 (0.50)	0.91 (0.51)	
Weight change during first-line therapy, n (%)	Loss <10 lbs	193 (42.79)	1663 (48.93)	.003
	Loss ≥10 lbs	113 (25.06)	659 (19.39)	
	Any weight gain	82 (18.18)	724 (21.30)	
	No change	12 (2.66)	61 (1.79)	
	Missing	51 (11.31)	292 (8.59)	
Number of metastatic sites	n	48	295	.10
	Mean (SD)	1.21 (0.582)	1.42 (0.857)	
Dose reduction during first-line therapy	Yes	56 (12.42)	547 (16.09)	.04
	No	395 (87.58)	2852 (83.91)	
Age	n	451	3399	.75
	Mean (SD)	66.4 (11.7)	66.2 (11.8)	
Disease status at advanced diagnosis, n (%)	Diagnosed metastatic	269 (59.65)	1769 (52.04)	.51
	Recurrent metastatic	49 (10.86)	359 (10.56)	
Primary tumor site. n (%)	Gastric	181 (40.13)	1207 (35.51)	.15
	GEJ	118 (26.16)	985 (28.98)	
	Esophageal	152 (33.70)	1207 (35.51)	
Gender, n (%)	Female	101 (22.39)	797 (23.45)	.62
	Male	350 (77.61)	2602 (76.55)	
Race, n (%)	White	307 (68.07)	2289 (67.34)	1.00
	Non-White	97 (21.51)	723 (21.27)	
Geographic region, n (%)	Northeast	98 (21.73)	707 (20.80)	.92
	South	151 (33.48)	1180 (34.72)	
	Midwest	79 (17.52)	582 (17.12)	
	West	79 (17.52)	620 (18.24)	

Abbreviations: SD, standard deviation; lbs, pounds; GEJ, gastroesophageal junction.

**Table 4.** Multivariable Analysis of Characteristics of Patients who did Versus did not Receive Second-Line Therapy.

Parameter	Value	Estimate	Standard Error	Wald Chi-Square	*P* Value	Effect	Odds Ratio	95% CI
Intercept		1.1779	0.3173	13.7851	.0002	Duration of first-line therapy		
Duration of first- line therapy	85-168 days	0.00285	0.1428	0.0004	.98	85-168 vs ≥169 days	1.003	0.76 -1.33
	≤84 days	−0.5696	0.1291	19.4778	<.0001	≤84 vs ≥169 days	0.57	0.44-0.73
Age	18-64 years	0.5429	0.0918	34.9426	<.0001	Age		
						18-64 vs 65+ years	1.72	1.44-2.06
Diagnosed metastatic	No	−0.7747	0.1024	57.249	<.0001	Diagnosed metastatic		
						No vs Yes	0.46	0.38-0.56
HER2 status	Negative	−0.3693	0.1588	5.4081	.0200	HER2 status		
	Missing/Unk	−0.3601	0.1536	5.4939	.0191	Unk/missing vs positive	0.70	0.52-0.94
						Negative vs positive	0.69	0.51-0.94
Weight loss during first-line therapy	Loss <10%	0.3419	0.1243	7.5654	.0059	Weight loss during first-line therapy		
	Weight gain	0.5814	0.1475	15.5327	<.0001	No change vs loss ≥ 10%	1.75	1.0-3.07
	No change	0.5575	0.2870	3.7737	.0521	Weight gain vs loss ≥ 10%	1.79	1.34-2.39
						Loss <10% vs ≥ 10%	1.41	1.10 -1.80

Abbreviation: CI, confidence interval; Unk, unknown.

## Discussion

Despite heterogeneous treatments as detailed in the Supplemental Appendix and consistent with prior research,^14^ receipt of second-line therapy was associated with improved overall survival. Although the reason for not receiving second line therapy is not evident in our data set and causal inference cannot be made, the findings demonstrate a statistically significant relationship between OS and second-line therapy.

The results from this study are consistent with data from prior clinical trials that have also demonstrated improved overall survival outcomes associated with additional lines of therapy in patients with gastroesophageal adenocarcinoma. The median survival for patients who received more than 1 line of therapy was approximately 6 months longer than that observed among patients who did not receive additional therapy. It is important to note that the exact magnitude of benefit cannot be directly compared with the randomized trials, in that the current analysis evaluated time from initiation of first-line therapy in order to appropriately balance on baseline covariates, whereas in the trials the survival time was only from the start of second-line therapy. Given that the duration of first-line therapy differed between the groups, this must also be taken into account when interpreting the magnitude of difference. Therefore, the difference of 6 months median survival time is for patients with first-line only versus first-line followed by subsequent therapy and represents the survival outcomes of a sequential approach to patient care, rather than independent lines of therapy. Of note, the crossover of the survival curves might imply that the patients who received first-line only were heterogeneous and could include some patients who responded and some who did not. Due to the nature of the EMR data, response and progression data are not available and this potential explanation for the crossover of the curves cannot be explored in this study. Given that this analysis was based on retrospective, nonrandomized data, these results, while consistent with what is known from clinical trial data, should be interpreted with caution.

Despite the limitations of retrospective analyses, these findings are consistent with the current sequential approach to therapy and survival results are highly consistent with the prior second-line clinical trials in gastroesophageal adenocarcinoma that have all demonstrated improved outcomes associated with continued systemic therapy versus best supportive care.^5,7,8^ The body of evidence is strengthened by real-world evidence supporting these trials in unselected patients. Of note, the regimens used for continued therapy cancer remain varied. The 2018 NCCN guidelines for gastric cancer indicate there is level 1 evidence for the preferred regimens ramucirumab plus paclitaxel, and for ramucirumab, irinotecan, docetaxel or paclitaxel monotherapy in the post-progression setting.^3,4^ A fluoropyrimidine plus irinotecan is also preferred with category 2A evidence. However, in this study, only 4 of the 7 most common regimens used in the second-line setting have been recommended with Category 2A evidence or higher in NCCN guidelines. Certainly, therapy may need to be individualized for unique clinical scenarios, but the relatively equal distribution of the most common second-line regimens observed in our data set (Table 2) reflects variability in real-world practice. The full list of regimens used for each line of therapy is presented in Supplemental Tables 1-3.

The use of evidence-based medicine may further improve the survival outcomes for gastroesophageal adenocarcinoma; however, the first-line treatment strategies observed in this study do not correspond to NCCN guidelines for preferred or level 1 evidence, so this could not be directly evaluated. There is a potential opportunity to further improve the survival outcomes of patients by ensuring that the treatment strategy considering subsequent lines of therapy is supported by evidence-based recommendations. Future research should evaluate the benefits of care concordant with NCCN guidelines in gastroesophageal carcinoma, as providers increasingly provide care that is supported by clinical trial data.

Importantly, the factors that may contribute to patients receiving subsequent care are critical. In this study, 451 (19.9%) patients were excluded from the survival analysis as if they died during first-line therapy, representing a much smaller proportion versus the 2516 patients that were alive and not continuing to receive therapy after first-line therapy. These patients can be partly accounted for by those with aggressive disease biology in which rapid cancer progression likely impacted the ability to receive further therapy. While the Flatiron database does not capture tumor genomic profiling given biomarker testing outside HER2 for first-line therapy remains experimental, certain oncogene amplifications such as *MET* and *FGFR2* have been identified in a minority of gastroesophageal cancers and portend poor prognosis.^15-17^ Such patients with poor prognostic biomarkers are likely underrepresented in randomized second-line trials, but were not excluded in this analysis, as the only tumor biomarker reported in the data set is HER2 status. Other factors may also play a role in the ability for patients to receive further therapy. Some of these factors could be evaluated in this study, such as performance status, body weight loss, and creatinine level. Others, such as patient choice for discontinuation or complex comorbid conditions, could not be evaluated due to the limitations of data fields contained in the available EMR data sets. While the impact of community versus academic practices on receipt of second-line therapy was not statistically significant (*P* > .01) and did not meet the stepwise selection criteria, the Flatiron data are more than 90% community-based practices, and the data set may not be appropriate to study this question due to the very small sample from academic practice settings.

In multivariable analysis, tumor HER2 positivity was associated with higher likelihood of receiving second-line therapy. HER2 overexpressing gastroesophageal cancers derive benefit from the addition of trastuzumab to first-line therapy. While hypothesis generating, improved categorization of biomarkers to develop successful molecularly targeted strategies to improve first-line treatment outcomes and increase the chance of receiving subsequent lines of therapy. Duration of first-line therapy was also found to be significant, which could be affected by improved clinical and supportive care but also could be associated with time to progressive disease. The available EMR data sets did not contain data on disease progression, so this could be a factor unaccounted for in the analysis. Importantly, patient weight loss during first-line therapy is a potentially modifiable factor that providers could address during first-line care to ensure patients maintain body weight during initial treatment. There is a need to further investigate this finding to understand whether the study of interventions to support patient body weight after advanced gastric cancer diagnosis are warranted.

Despite the limitations of retrospective analyses with regard to causal inference, this study provides evidence regarding a potential association between survival outcomes and therapy after first-line treatment among patients able to receive subsequent treatment. There is a need to ensure patients receive the best possible outcomes of treatment by providing category 1 evidence-based care whenever possible and to consider taking a sequential approach to therapy to ensure as many patients as possible remain well enough to continue therapy if and when first-line therapy fails.

## Supplemental Material

SUPPLEMENTAL_TABLES - Real-World Outcomes and Factors Associated With the Second-Line Treatment of Patients With Gastric, Gastroesophageal Junction, or Esophageal Adenocarcinoma Click here for additional data file. SUPPLEMENTAL_TABLES for Real-World Outcomes and Factors Associated With the Second-Line Treatment of Patients With Gastric, Gastroesophageal Junction, or Esophageal Adenocarcinoma by Afsaneh Barzi, Lisa M. Hess, Yajun E. Zhu, Astra M. Liepa, Tomoko Sugihara, Julie Beyrer and Joseph Chao in Cancer Control
