# Supplementary material for: Real-World Outcomes and Factors Associated With the Second-Line Treatment of Patients With Gastric, Gastroesophageal Junction, or Esophageal Adenocarcinoma
Source: Cancer Control. 2019 May 6;26(1):1073274819847642. doi: 10.1177/1073274819847642 (PMC6503607; doi:10.1177/1073274819847642)
Supplement: SUPPLEMENTAL_TABLES - Real-World Outcomes and Factors Associated With the Second-Line Treatment of Patients With Gastric, Gastroesophageal Junction, or Esophageal Adenocarcinoma [file SUPPLEMENTAL_TABLES.pdf]

Supplemental Table 1. Regimens received by patients with gastric tumors

Regimen , n(%)	Line 1 (N=1388)	Line 2 (N=530)	Line 3 (N=217)
Abiraterone,Cisplatin,Irinotecan	0 (0.00)	0 (0.00)	1 (0.46)
Atezolizumab	0 (0.00)	1 (0.19)	0 (0.00)
Capecitabine,Anastrozole	0 (0.00)	1 (0.19)	0 (0.00)
Capecitabine,Carboplatin,Paclitaxel Protein-Bound	0 (0.00)	0 (0.00)	0 (0.00)
Capecitabine,Cisplatin,Docetaxel, Trastuzumab	0 (0.00)	1 (0.19)	0 (0.00)
Capecitabine,Cisplatin,Paclitaxel	0 (0.00)	1 (0.19)	0 (0.00)
Capecitabine,Epirubicin	0 (0.00)	0 (0.00)	2 (0.92)
Capecitabine,Paclitaxel	0 (0.00)	2 (0.38)	0 (0.00)
Capecitabine,Paclitaxel,Ramucirumab	0 (0.00)	1 (0.19)	0 (0.00)
Capecitabine,Paclitaxel,Trastuzumab	0 (0.00)	0 (0.00)	1 (0.46)
Capecitabine,Pembrolizumab	0 (0.00)	1 (0.19)	1 (0.46)
Carboplatin,Clinical Study Drug	0 (0.00)	1 (0.19)	0 (0.00)
Carboplatin,Docetaxel,Paclitaxel,Ramucirumab	0 (0.00)	1 (0.19)	0 (0.00)
Carboplatin,Docetaxel,Ramucirumab	0 (0.00)	2 (0.38)	0 (0.00)
Carboplatin,Trastuzumab	0 (0.00)	1 (0.19)	0 (0.00)
Cisplatin,Dabrafenib,Ramucirumab, Trametinib	0 (0.00)	1 (0.19)	0 (0.00)
Cisplatin,Doxorubicin,Etoposide	0 (0.00)	0 (0.00)	0 (0.00)
Cisplatin,Paclitaxel	0 (0.00)	2 (0.38)	0 (0.00)
Clinical Study Drug,Irinotecan,Ramucirumab	0 (0.00)	1 (0.19)	0 (0.00)
Clinical Study Drug,Paclitaxel,Ramucirumab	0 (0.00)	1 (0.19)	0 (0.00)
Clinical Study Drug,Ramucirumab	0 (0.00)	2 (0.38)	0 (0.00)
Crizotinib,Irinotecan,Cetuximab	0 (0.00)	0 (0.00)	1 (0.46)
Docetaxel,Gemcitabine,Irinotecan,Oxaliplatin, Trastuzumab	0 (0.00)	1 (0.19)	0 (0.00)
Docetaxel,Ramucirumab	0 (0.00)	4 (0.75)	5 (2.30)
Etoposide,Irinotecan,Oxaliplatin,Bevacizumab	0 (0.00)	0 (0.00)	1 (0.46)
Everolimus,Ramucirumab	0 (0.00)	0 (0.00)	0 (0.00)
FOLFIRI,Ramucirumab	0 (0.00)	0 (0.00)	1 (0.46)
Fluorouracil,Carboplatin	0 (0.00)	2 (0.38)	0 (0.00)

Fluorouracil,Carboplatin,Epirubicin,Ramucirumab	0 (0.00)	1 (0.19)	0 (0.00)
Fluorouracil,Carboplatin,Irinotecan,Leucovorin,Paclitaxel	0 (0.00)	1 (0.19)	0 (0.00)
Fluorouracil,Carboplatin,Paclitaxel,Bevacizumab	0 (0.00)	1 (0.19)	0 (0.00)
Fluorouracil,Cisplatin,Docetaxel,Leucovorin,Paclitaxel,Ramucirumab	0 (0.00)	1 (0.19)	0 (0.00)
Fluorouracil,Cisplatin,Docetaxel,Oxaliplatin	0 (0.00)	1 (0.19)	0 (0.00)
Fluorouracil,Cisplatin,Docetaxel,Pembrolizumab	0 (0.00)	1 (0.19)	0 (0.00)
Fluorouracil,Docetaxel,Leucovorin	0 (0.00)	1 (0.19)	0 (0.00)
Fluorouracil,Doxorubicin,Mitomycin	0 (0.00)	1 (0.19)	0 (0.00)
Fluorouracil,Etoposide,Leucovorin	0 (0.00)	1 (0.19)	0 (0.00)
Fluorouracil,Etoposide,Leucovorin, Trastuzumab	0 (0.00)	1 (0.19)	0 (0.00)
Fluorouracil,Leucovorin,Leuprolide,Oxaliplatin	0 (0.00)	0 (0.00)	1 (0.46)
Fluorouracil,Leucovorin,Oxaliplatin,Trastuzumab, Triptorelin	0 (0.00)	1 (0.19)	0 (0.00)
Fluorouracil,Nivolumab,Oxaliplatin	0 (0.00)	1 (0.19)	0 (0.00)
Fluorouracil,Oxaliplatin,Paclitaxel,Bevacizumab	0 (0.00)	1 (0.19)	0 (0.00)
Fluorouracil,Paclitaxel Protein-Bound,Bevacizumab	0 (0.00)	0 (0.00)	1 (0.46)
Fluorouracil,Paclitaxel,Ramucirumab	0 (0.00)	1 (0.19)	0 (0.00)
Irinotecan,Oxaliplatin,Trastuzumab	0 (0.00)	0 (0.00)	0 (0.00)
Irinotecan,Paclitaxel,Ramucirumab	0 (0.00)	0 (0.00)	1 (0.46)
Irinotecan,Ramucirumab	0 (0.00)	1 (0.19)	0 (0.00)
Irinotecan,Trastuzumab	0 (0.00)	0 (0.00)	2 (0.92)
Medroxyprogesterone,Pembrolizumab	0 (0.00)	1 (0.19)	0 (0.00)
Methotrexate	0 (0.00)	1 (0.19)	0 (0.00)
Oxaliplatin,Bevacizumab	0 (0.00)	2 (0.38)	0 (0.00)
Paclitaxel Protein-Bound	0 (0.00)	2 (0.38)	1 (0.46)
Paclitaxel,Pembrolizumab,Ramucirumab	0 (0.00)	0 (0.00)	0 (0.00)
Paclitaxel,Ramucirumab,Regorafenib	0 (0.00)	1 (0.19)	0 (0.00)
Paclitaxel,Ramucirumab,Trastuzumab	0 (0.00)	1 (0.19)	0 (0.00)
Pertuzumab,Trastuzumab	0 (0.00)	0 (0.00)	1 (0.46)
Ramucirumab,Trastuzumab	0 (0.00)	1 (0.19)	0 (0.00)
Sunitinib	0 (0.00)	0 (0.00)	1 (0.46)
Topotecan	0 (0.00)	1 (0.19)	0 (0.00)

Anastrozole	1 (0.07)	0 (0.00)	0 (0.00)
Bicalutamide,Triptorelin	1 (0.07)	0 (0.00)	0 (0.00)
Bortezomib	1 (0.07)	0 (0.00)	0 (0.00)
Busulfan	1 (0.07)	0 (0.00)	0 (0.00)
CAPEOX,Bevacizumab	1 (0.07)	0 (0.00)	0 (0.00)
Capecitabine,Carboplatin,Docetaxel	1 (0.07)	0 (0.00)	0 (0.00)
Capecitabine,Carboplatin,Epirubicin,Oxaliplatin	1 (0.07)	0 (0.00)	0 (0.00)
Capecitabine,Carboplatin,Paclitaxel,Bevacizumab	1 (0.07)	1 (0.19)	0 (0.00)
Capecitabine,Carboplatin,Paclitaxel,Trastuzumab	1 (0.07)	1 (0.19)	0 (0.00)
Capecitabine,Carboplatin, Trastuzumab	1 (0.07)	0 (0.00)	0 (0.00)
Capecitabine,Cisplatin,Clinical Study Drug	1 (0.07)	0 (0.00)	1 (0.46)
Capecitabine,Cisplatin,Epirubicin,Leucovorin,Oxaliplatin	1 (0.07)	0 (0.00)	0 (0.00)
Capecitabine,Clinical Study Drug	1 (0.07)	0 (0.00)	0 (0.00)
Capecitabine,Docetaxel,Epirubicin,Oxaliplatin	1 (0.07)	0 (0.00)	0 (0.00)
Capecitabine,Docetaxel,Oxaliplatin, Trastuzumab	1 (0.07)	0 (0.00)	0 (0.00)
Capecitabine,Epirubicin,Leucovorin,Oxaliplatin	1 (0.07)	1 (0.19)	0 (0.00)
Capecitabine,Irinotecan	1 (0.07)	3 (0.57)	0 (0.00)
Capecitabine,Irinotecan,Oxaliplatin	1 (0.07)	0 (0.00)	0 (0.00)
Capecitabine,Leuprolide,Oxaliplatin	1 (0.07)	0 (0.00)	0 (0.00)
Capecitabine,Ramucirumab	1 (0.07)	1 (0.19)	3 (1.38)
Carboplatin	1 (0.07)	0 (0.00)	0 (0.00)
Carboplatin,Docetaxel,Pembrolizumab,Ramucirumab	1 (0.07)	0 (0.00)	0 (0.00)
Carboplatin,Etoposide,Trastuzumab	1 (0.07)	0 (0.00)	0 (0.00)
Carboplatin,Pembrolizumab,Pemetrexed	1 (0.07)	0 (0.00)	0 (0.00)
Cisplatin	1 (0.07)	1 (0.19)	0 (0.00)
Cisplatin,Clinical Study Drug,Docetaxel	1 (0.07)	0 (0.00)	0 (0.00)
Cisplatin,Docetaxel,Irinotecan	1 (0.07)	0 (0.00)	0 (0.00)
Cisplatin,Docetaxel,Leucovorin	1 (0.07)	0 (0.00)	0 (0.00)
Cisplatin,Etoposide	1 (0.07)	0 (0.00)	0 (0.00)
Cisplatin,Gemcitabine	1 (0.07)	0 (0.00)	0 (0.00)
Cisplatin,Irinotecan,Trastuzumab	1 (0.07)	0 (0.00)	2 (0.92)

Cyclophosphamide,Doxorubicin,Rituximab,Vincristine	1 (0.07)	0 (0.00)	0 (0.00)
Docetaxel,Irinotecan	1 (0.07)	3 (0.57)	3 (1.38)
Enzalutamide	1 (0.07)	0 (0.00)	0 (0.00)
Erlotinib	1 (0.07)	0 (0.00)	0 (0.00)
FOLFOX,Ramucirumab	1 (0.07)	0 (0.00)	0 (0.00)
Fluorouracil,Abiraterone,Leucovorin,Oxaliplatin	1 (0.07)	1 (0.19)	0 (0.00)
Fluorouracil,Carboplatin,Docetaxel,Leucovorin,Oxaliplatin	1 (0.07)	0 (0.00)	0 (0.00)
Fluorouracil,Carboplatin,Docetaxel,Paclitaxel	1 (0.07)	0 (0.00)	0 (0.00)
Fluorouracil,Carboplatin,Epirubicin	1 (0.07)	1 (0.19)	0 (0.00)
Fluorouracil,Carboplatin,Leucovorin	1 (0.07)	0 (0.00)	0 (0.00)
Fluorouracil,Carboplatin,Leucovorin,Oxaliplatin	1 (0.07)	0 (0.00)	0 (0.00)
Fluorouracil,Carboplatin,Trastuzumab	1 (0.07)	1 (0.19)	0 (0.00)
Fluorouracil,Cisplatin,Clinical Study Drug	1 (0.07)	0 (0.00)	0 (0.00)
Fluorouracil,Cisplatin,Epirubicin,Leucovorin	1 (0.07)	0 (0.00)	0 (0.00)
Fluorouracil,Cisplatin,Epirubicin,Leucovorin,Oxaliplatin	1 (0.07)	0 (0.00)	0 (0.00)
Fluorouracil,Cisplatin,Gemcitabine,Leucovorin,Mitomycin	1 (0.07)	0 (0.00)	0 (0.00)
Fluorouracil,Clinical Study Drug,Oxaliplatin	1 (0.07)	0 (0.00)	0 (0.00)
Fluorouracil,Docetaxel,Epirubicin,Oxaliplatin	1 (0.07)	0 (0.00)	0 (0.00)
Fluorouracil,Epirubicin,Oxaliplatin,Sorafenib	1 (0.07)	0 (0.00)	0 (0.00)
Fluorouracil,Gemcitabine	1 (0.07)	0 (0.00)	0 (0.00)
Fluorouracil,Gemcitabine,Leucovorin,Oxaliplatin,Paclitaxel Protein-Bound	1 (0.07)	0 (0.00)	0 (0.00)
Fluorouracil,Leucovorin,Bevacizumab	1 (0.07)	0 (0.00)	0 (0.00)
Fluorouracil,Leucovorin,Oxaliplatin,Paclitaxel,Ramucirumab	1 (0.07)	0 (0.00)	0 (0.00)
Fluorouracil,Leucovorin,Oxaliplatin,Pembrolizumab	1 (0.07)	1 (0.19)	0 (0.00)
Fluorouracil,Leucovorin,Trastuzumab	1 (0.07)	2 (0.38)	1 (0.46)
Fluorouracil,Trastuzumab	1 (0.07)	1 (0.19)	0 (0.00)
Fulvestrant	1 (0.07)	0 (0.00)	0 (0.00)
Gemcitabine	1 (0.07)	1 (0.19)	0 (0.00)
Gemcitabine,Irinotecan	1 (0.07)	0 (0.00)	0 (0.00)
Gemcitabine,Paclitaxel Protein-Bound	1 (0.07)	0 (0.00)	0 (0.00)
Irinotecan,Mitomycin	1 (0.07)	0 (0.00)	0 (0.00)

Leuprolide	1 (0.07)	0 (0.00)	0 (0.00)
Medroxyprogesterone	1 (0.07)	0 (0.00)	0 (0.00)
Oxaliplatin,Trastuzumab	1 (0.07)	0 (0.00)	0 (0.00)
Paclitaxel Protein-Bound,Trastuzumab	1 (0.07)	0 (0.00)	0 (0.00)
Paclitaxel,Pemetrexed	1 (0.07)	0 (0.00)	0 (0.00)
Paclitaxel,Trastuzumab	1 (0.07)	4 (0.75)	0 (0.00)
Rituximab	1 (0.07)	0 (0.00)	0 (0.00)
Sipuleucel-T	1 (0.07)	0 (0.00)	0 (0.00)
Triptorelin	1 (0.07)	0 (0.00)	0 (0.00)
Fluorouracil,Carboplatin,Docetaxel	10 (0.72)	1 (0.19)	0 (0.00)
Carboplatin,Docetaxel	12 (0.86)	2 (0.38)	1 (0.46)
Fluorouracil,Cisplatin,Trastuzumab	12 (0.86)	1 (0.19)	1 (0.46)
Capecitabine	120 (8.65)	24 (4.53)	7 (3.23)
Ramucirumab	13 (0.94)	32 (6.04)	23 (10.60)
Capecitabine,Cisplatin,Epirubicin	14 (1.01)	1 (0.19)	0 (0.00)
Clinical Study Drug	15 (1.08)	11 (2.08)	10 (4.61)
Fluorouracil,Cisplatin,Docetaxel,Leucovorin	15 (1.08)	2 (0.38)	1 (0.46)
Fluorouracil	16 (1.15)	2 (0.38)	1 (0.46)
Fluorouracil,Cisplatin	17 (1.22)	3 (0.57)	0 (0.00)
Fluorouracil,Epirubicin,Oxaliplatin	18 (1.30)	2 (0.38)	0 (0.00)
Azacitidine	2 (0.14)	0 (0.00)	0 (0.00)
Capecitabine,Cisplatin,Docetaxel	2 (0.14)	2 (0.38)	0 (0.00)
Capecitabine,Leucovorin	2 (0.14)	0 (0.00)	0 (0.00)
Carboplatin,Gemcitabine	2 (0.14)	0 (0.00)	0 (0.00)
Carboplatin,Irinotecan	2 (0.14)	1 (0.19)	1 (0.46)
Cisplatin,Docetaxel,Trastuzumab	2 (0.14)	0 (0.00)	0 (0.00)
Cisplatin,Epirubicin	2 (0.14)	0 (0.00)	0 (0.00)
Docetaxel,Oxaliplatin	2 (0.14)	0 (0.00)	0 (0.00)
Fluorouracil,Carboplatin,Docetaxel,Leucovorin	2 (0.14)	0 (0.00)	0 (0.00)
Fluorouracil,Carboplatin,Leucovorin,Oxaliplatin,Paclitaxel	2 (0.14)	0 (0.00)	0 (0.00)
Fluorouracil,Cisplatin,Docetaxel,Leucovorin,Trastuzumab	2 (0.14)	0 (0.00)	0 (0.00)

Fluorouracil,Cisplatin,Docetaxel,Trastuzumab	2 (0.14)	0 (0.00)	0 (0.00)
Fluorouracil,Epirubicin,Leucovorin,Oxaliplatin	2 (0.14)	2 (0.38)	0 (0.00)
Letrozole	2 (0.14)	0 (0.00)	1 (0.46)
Tamoxifen	2 (0.14)	0 (0.00)	0 (0.00)
Fluorouracil,Docetaxel,Leucovorin,Oxaliplatin	20 (1.44)	1 (0.19)	1 (0.46)
Capecitabine,Carboplatin,Paclitaxel	22 (1.59)	2 (0.38)	1 (0.46)
Carboplatin,Paclitaxel	23 (1.66)	10 (1.89)	3 (1.38)
FOLFIRI	24 (1.73)	34 (6.42)	20 (9.22)
Fluorouracil,Docetaxel,Oxaliplatin	28 (2.02)	2 (0.38)	0 (0.00)
Capecitabine,Carboplatin	3 (0.22)	0 (0.00)	1 (0.46)
Capecitabine,Docetaxel	3 (0.22)	1 (0.19)	0 (0.00)
Carboplatin,Etoposide	3 (0.22)	0 (0.00)	0 (0.00)
Carboplatin,Paclitaxel Protein-Bound	3 (0.22)	1 (0.19)	0 (0.00)
FOLFIRI,Trastuzumab	3 (0.22)	2 (0.38)	3 (1.38)
Fluorouracil,Clinical Study Drug,Leucovorin,Oxaliplatin	3 (0.22)	0 (0.00)	0 (0.00)
Fluorouracil,Docetaxel,Oxaliplatin, Trastuzumab	3 (0.22)	1 (0.19)	0 (0.00)
Nivolumab	3 (0.22)	5 (0.94)	12 (5.53)
Fluorouracil,Leucovorin	32 (2.31)	8 (1.51)	1 (0.46)
Paclitaxel,Ramucirumab	32 (2.31)	107 (20.19)	37 (17.05)
FOLFOX	376 (27.09)	62 (11.70)	14 (6.45)
CAPEOX,Trastuzumab	4 (0.29)	2 (0.38)	0 (0.00)
Carboplatin,Paclitaxel,Ramucirumab	4 (0.29)	0 (0.00)	0 (0.00)
FOLFOX,Bevacizumab	4 (0.29)	2 (0.38)	0 (0.00)
Paclitaxel	4 (0.29)	9 (1.70)	3 (1.38)
FOLFOX,Trastuzumab	47 (3.39)	8 (1.51)	0 (0.00)
Capecitabine,Cisplatin,Trastuzumab	5 (0.36)	2 (0.38)	0 (0.00)
Capecitabine,Trastuzumab	5 (0.36)	3 (0.57)	1 (0.46)
Carboplatin,Docetaxel,Trastuzumab	5 (0.36)	1 (0.19)	2 (0.92)
Cisplatin,Docetaxel	5 (0.36)	1 (0.19)	1 (0.46)
Cisplatin,Trastuzumab	5 (0.36)	1 (0.19)	0 (0.00)
Docetaxel,Trastuzumab	5 (0.36)	6 (1.13)	2 (0.92)

Fluorouracil,Carboplatin,Paclitaxel	5 (0.36)	0 (0.00)	0 (0.00)
Fluorouracil,Cisplatin,Leucovorin	5 (0.36)	1 (0.19)	0 (0.00)
Irinotecan	5 (0.36)	7 (1.32)	15 (6.91)
Pembrolizumab	5 (0.36)	10 (1.89)	8 (3.69)
CAPEOX	50 (3.60)	10 (1.89)	2 (0.92)
Fluorouracil,Cisplatin,Docetaxel	50 (3.60)	5 (0.94)	0 (0.00)
Carboplatin,Paclitaxel,Radiation	52 (3.75)	21 (3.96)	4 (1.84)
Fluorouracil,Cisplatin,Epirubicin	52 (3.75)	2 (0.38)	2 (0.92)
Carboplatin,Paclitaxel,Trastuzumab	6 (0.43)	1 (0.19)	1 (0.46)
Cisplatin,Irinotecan	6 (0.43)	4 (0.75)	2 (0.92)
Epirubicin,Oxaliplatin	6 (0.43)	2 (0.38)	0 (0.00)
Oxaliplatin	6 (0.43)	4 (0.75)	1 (0.46)
Capecitabine,Cisplatin	7 (0.50)	1 (0.19)	0 (0.00)
Docetaxel	7 (0.50)	20 (3.77)	1 (0.46)
FOLFOXIRI	7 (0.50)	1 (0.19)	0 (0.00)
Capecitabine,Epirubicin,Oxaliplatin	74 (5.33)	5 (0.94)	1 (0.46)
Trastuzumab	8 (0.58)	4 (0.75)	1 (0.46)

Supplemental Table 2. Regimens received by patients with gastroesophageal junction tumors

Regimen , n(%)	Line 1 (N=1103)	Line 2 (N=502)	Line 3 (N=232)
Ado-Trastuzumab Emtansine,Ramucirumab	0 (0.00)	0 (0.00)	1 (0.43)
Anastrozole,Carboplatin,Paclitaxel	1 (0.09)	0 (0.00)	0 (0.00)
Azacitidine	0 (0.00)	0 (0.00)	1 (0.43)
Bcg Vaccine	0 (0.00)	1 (0.20)	0 (0.00)
Bendamustine,Rituximab,Rituximab/Hyaluronidase	0 (0.00)	1 (0.20)	0 (0.00)
Bicalutamide,Carboplatin,Leuprolide,Paclitaxel	1 (0.09)	0 (0.00)	0 (0.00)
CAPEOX	17 (1.54)	8 (1.59)	5 (2.16)
CAPEOX,Trastuzumab	6 (0.54)	1 (0.20)	0 (0.00)

Capecitabine	39 (3.54)	24 (4.78)	4 (1.72)
Capecitabine,Atezolizumab,Leucovorin,Oxaliplatin	1 (0.09)	0 (0.00)	0 (0.00)
Capecitabine,Bevacizumab	0 (0.00)	0 (0.00)	1 (0.43)
Capecitabine,Carboplatin	1 (0.09)	0 (0.00)	0 (0.00)
Capecitabine,Carboplatin,Docetaxel,Paclitaxel	1 (0.09)	1 (0.20)	0 (0.00)
Capecitabine,Carboplatin,Leucovorin,Oxaliplatin,Paclitaxel	1 (0.09)	0 (0.00)	0 (0.00)
Capecitabine,Carboplatin,Paclitaxel	6 (0.54)	0 (0.00)	0 (0.00)
Capecitabine,Carboplatin,Paclitaxel Protein-Bound	2 (0.18)	1 (0.20)	0 (0.00)
Capecitabine,Carboplatin,Paclitaxel,Ramucirumab	0 (0.00)	1 (0.20)	0 (0.00)
Capecitabine,Carboplatin, Trastuzumab	2 (0.18)	1 (0.20)	0 (0.00)
Capecitabine,Cisplatin	2 (0.18)	1 (0.20)	0 (0.00)
Capecitabine,Cisplatin,Clinical Study Drug	2 (0.18)	0 (0.00)	0 (0.00)
Capecitabine,Cisplatin,Doxorubicin	0 (0.00)	1 (0.20)	0 (0.00)
Capecitabine,Cisplatin,Epirubicin	4 (0.36)	0 (0.00)	0 (0.00)
Capecitabine,Cisplatin,Epirubicin,Oxaliplatin, Trastuzumab	2 (0.18)	0 (0.00)	0 (0.00)
Capecitabine,Cisplatin,Trastuzumab	11 (1.00)	2 (0.40)	0 (0.00)
Capecitabine,Clinical Study Drug	3 (0.27)	1 (0.20)	0 (0.00)
Capecitabine,Docetaxel	3 (0.27)	1 (0.20)	2 (0.86)
Capecitabine,Docetaxel,Irinotecan,Paclitaxel	0 (0.00)	1 (0.20)	0 (0.00)
Capecitabine,Docetaxel,Oxaliplatin	1 (0.09)	0 (0.00)	0 (0.00)
Capecitabine,Epirubicin	0 (0.00)	0 (0.00)	0 (0.00)
Capecitabine,Epirubicin,Leucovorin,Oxaliplatin	1 (0.09)	0 (0.00)	0 (0.00)
Capecitabine,Epirubicin,Oxaliplatin	33 (2.99)	9 (1.79)	2 (0.86)
Capecitabine,Epirubicin,Oxaliplatin, Tamoxifen	0 (0.00)	1 (0.20)	0 (0.00)
Capecitabine,Epirubicin,Oxaliplatin, Trastuzumab	0 (0.00)	1 (0.20)	0 (0.00)
Capecitabine,Gemcitabine	1 (0.09)	0 (0.00)	1 (0.43)
Capecitabine,Irinotecan	0 (0.00)	1 (0.20)	2 (0.86)
Capecitabine,Lapatinib	0 (0.00)	1 (0.20)	0 (0.00)
Capecitabine,Paclitaxel	0 (0.00)	0 (0.00)	2 (0.86)
Capecitabine,Paclitaxel,Trastuzumab	0 (0.00)	1 (0.20)	0 (0.00)
Capecitabine,Ramucirumab	0 (0.00)	1 (0.20)	1 (0.43)

Capecitabine,Trastuzumab	6 (0.54)	5 (1.00)	1 (0.43)
Carboplatin	5 (0.45)	0 (0.00)	0 (0.00)
Carboplatin,Clinical Study Drug,Docetaxel	0 (0.00)	0 (0.00)	1 (0.43)
Carboplatin,Docetaxel	14 (1.27)	5 (1.00)	2 (0.86)
Carboplatin,Docetaxel,Trastuzumab	1 (0.09)	2 (0.40)	0 (0.00)
Carboplatin,Etoposide	0 (0.00)	2 (0.40)	0 (0.00)
Carboplatin,Irinotecan	2 (0.18)	1 (0.20)	0 (0.00)
Carboplatin,Irinotecan,Paclitaxel,Ramucirumab,Trastuzumab	0 (0.00)	1 (0.20)	0 (0.00)
Carboplatin,Irinotecan,Ramucirumab	1 (0.09)	0 (0.00)	0 (0.00)
Carboplatin,Irinotecan,Trastuzumab	0 (0.00)	2 (0.40)	0 (0.00)
Carboplatin,Paclitaxel	56 (5.08)	10 (1.99)	4 (1.72)
Carboplatin,Paclitaxel Protein-Bound	2 (0.18)	0 (0.00)	0 (0.00)
Carboplatin,Paclitaxel Protein-Bound,Trastuzumab	0 (0.00)	1 (0.20)	0 (0.00)
Carboplatin,Paclitaxel,Bevacizumab	0 (0.00)	0 (0.00)	1 (0.43)
Carboplatin,Paclitaxel,Pemetrexed	1 (0.09)	0 (0.00)	0 (0.00)
Carboplatin,Paclitaxel,Radiation	263 (23.84)	16 (3.19)	5 (2.16)
Carboplatin,Paclitaxel,Ramucirumab	0 (0.00)	1 (0.20)	2 (0.86)
Carboplatin,Paclitaxel,Trastuzumab	19 (1.72)	12 (2.39)	3 (1.29)
Carboplatin,Trastuzumab	1 (0.09)	1 (0.20)	0 (0.00)
Cetuximab	0 (0.00)	0 (0.00)	1 (0.43)
Cisplatin	3 (0.27)	1 (0.20)	0 (0.00)
Cisplatin,Docetaxel	7 (0.63)	0 (0.00)	0 (0.00)
Cisplatin,Docetaxel,Trastuzumab	1 (0.09)	0 (0.00)	0 (0.00)
Cisplatin,Doxorubicin,Paclitaxel	0 (0.00)	0 (0.00)	1 (0.43)
Cisplatin,Etoposide	1 (0.09)	0 (0.00)	0 (0.00)
Cisplatin,Gemcitabine	2 (0.18)	0 (0.00)	0 (0.00)
Cisplatin,Irinotecan	4 (0.36)	10 (1.99)	7 (3.02)
Cisplatin,Paclitaxel	2 (0.18)	2 (0.40)	0 (0.00)
Cisplatin,Trastuzumab	0 (0.00)	0 (0.00)	1 (0.43)
Clinical Study Drug	18 (1.63)	9 (1.79)	10 (4.31)
Clinical Study Drug,Leucovorin	0 (0.00)	1 (0.20)	0 (0.00)

Clinical Study Drug,Ramucirumab	1 (0.09)	0 (0.00)	0 (0.00)
Docetaxel	5 (0.45)	9 (1.79)	7 (3.02)
Docetaxel,Doxorubicin	0 (0.00)	1 (0.20)	0 (0.00)
Docetaxel,Irinotecan	2 (0.18)	9 (1.79)	0 (0.00)
Docetaxel,Oxaliplatin,Trastuzumab	0 (0.00)	1 (0.20)	0 (0.00)
Docetaxel,Paclitaxel,Ramucirumab	0 (0.00)	0 (0.00)	1 (0.43)
Docetaxel,Ramucirumab	0 (0.00)	0 (0.00)	2 (0.86)
Docetaxel,Trastuzumab	0 (0.00)	7 (1.39)	2 (0.86)
Epirubicin,Oxaliplatin	1 (0.09)	1 (0.20)	1 (0.43)
Epirubicin,Oxaliplatin,Trastuzumab	1 (0.09)	0 (0.00)	1 (0.43)
Epirubicin,Paclitaxel	0 (0.00)	0 (0.00)	1 (0.43)
Everolimus,Paclitaxel,Ramucirumab	0 (0.00)	0 (0.00)	1 (0.43)
Exemestane	0 (0.00)	1 (0.20)	0 (0.00)
FOLFIRI	18 (1.63)	30 (5.98)	21 (9.05)
FOLFIRI,Bevacizumab	0 (0.00)	0 (0.00)	1 (0.43)
FOLFIRI,Ramucirumab	0 (0.00)	2 (0.40)	1 (0.43)
FOLFIRI,Trastuzumab	2 (0.18)	7 (1.39)	2 (0.86)
FOLFOX	215 (19.49)	65 (12.95)	12 (5.17)
FOLFOX,Bevacizumab	3 (0.27)	0 (0.00)	0 (0.00)
FOLFOX,Ramucirumab	1 (0.09)	0 (0.00)	1 (0.43)
FOLFOX,Trastuzumab	53 (4.81)	19 (3.78)	5 (2.16)
FOLFOX,Trastuzumab,Bevacizumab	1 (0.09)	0 (0.00)	0 (0.00)
FOLFOXIRI	0 (0.00)	1 (0.20)	1 (0.43)
FOLFOXIRI,Bevacizumab	0 (0.00)	0 (0.00)	0 (0.00)
Floxuridine	0 (0.00)	1 (0.20)	0 (0.00)
Fluorouracil	13 (1.18)	2 (0.40)	0 (0.00)
Fluorouracil,Amifostine,Leucovorin,Oxaliplatin,Trastuzumab	1 (0.09)	0 (0.00)	0 (0.00)
Fluorouracil,Anastrozole,Leucovorin,Oxaliplatin	0 (0.00)	1 (0.20)	0 (0.00)
Fluorouracil,Carboplatin	5 (0.45)	1 (0.20)	0 (0.00)
Fluorouracil,Carboplatin,Docetaxel	3 (0.27)	0 (0.00)	1 (0.43)
Fluorouracil,Carboplatin,Docetaxel,Trastuzumab	1 (0.09)	1 (0.20)	0 (0.00)

Fluorouracil,Carboplatin,Etoposide,Irinotecan,Leucovorin	0 (0.00)	1 (0.20)	0 (0.00)
Fluorouracil,Carboplatin,Leucovorin,Oxaliplatin,Paclitaxel	7 (0.63)	0 (0.00)	0 (0.00)
Fluorouracil,Carboplatin,Leucovorin,Oxaliplatin,Paclitaxel,Trastuzumab	2 (0.18)	0 (0.00)	0 (0.00)
Fluorouracil,Carboplatin,Leucovorin,Paclitaxel	1 (0.09)	0 (0.00)	0 (0.00)
Fluorouracil,Carboplatin,Paclitaxel	7 (0.63)	0 (0.00)	0 (0.00)
Fluorouracil,Carboplatin,Paclitaxel Protein-Bound	0 (0.00)	1 (0.20)	0 (0.00)
Fluorouracil,Carboplatin,Paclitaxel Protein-Bound,Ramucirumab	0 (0.00)	0 (0.00)	1 (0.43)
Fluorouracil,Carboplatin,Paclitaxel,Trastuzumab	0 (0.00)	0 (0.00)	1 (0.43)
Fluorouracil,Carboplatin,Trastuzumab	2 (0.18)	0 (0.00)	0 (0.00)
Fluorouracil,Cisplatin	24 (2.18)	3 (0.60)	0 (0.00)
Fluorouracil,Cisplatin,Clinical Study Drug,Trastuzumab	2 (0.18)	0 (0.00)	0 (0.00)
Fluorouracil,Cisplatin,Docetaxel	27 (2.45)	3 (0.60)	0 (0.00)
Fluorouracil,Cisplatin,Docetaxel,Gemcitabine	1 (0.09)	0 (0.00)	0 (0.00)
Fluorouracil,Cisplatin,Docetaxel,Leucovorin	8 (0.73)	2 (0.40)	0 (0.00)
Fluorouracil,Cisplatin,Docetaxel,Leucovorin,Trastuzumab	3 (0.27)	1 (0.20)	0 (0.00)
Fluorouracil,Cisplatin,Docetaxel,Oxaliplatin	1 (0.09)	0 (0.00)	0 (0.00)
Fluorouracil,Cisplatin,Docetaxel,Oxaliplatin,Trastuzumab	1 (0.09)	0 (0.00)	0 (0.00)
Fluorouracil,Cisplatin,Docetaxel,Paclitaxel	1 (0.09)	0 (0.00)	0 (0.00)
Fluorouracil,Cisplatin,Docetaxel,Pembrolizumab	1 (0.09)	0 (0.00)	0 (0.00)
Fluorouracil,Cisplatin,Docetaxel,Trastuzumab	2 (0.18)	0 (0.00)	0 (0.00)
Fluorouracil,Cisplatin,Epirubicin	21 (1.90)	6 (1.20)	0 (0.00)
Fluorouracil,Cisplatin,Gemcitabine,Leucovorin,Oxaliplatin	1 (0.09)	0 (0.00)	0 (0.00)
Fluorouracil,Cisplatin,Irinotecan,Leucovorin	1 (0.09)	0 (0.00)	1 (0.43)
Fluorouracil,Cisplatin,Irinotecan,Leucovorin,Oxaliplatin	0 (0.00)	0 (0.00)	0 (0.00)
Fluorouracil,Cisplatin,Leucovorin	1 (0.09)	2 (0.40)	0 (0.00)
Fluorouracil,Cisplatin,Leucovorin,Oxaliplatin	1 (0.09)	0 (0.00)	0 (0.00)
Fluorouracil,Cisplatin,Leucovorin,Trastuzumab	1 (0.09)	1 (0.20)	0 (0.00)
Fluorouracil,Cisplatin,Paclitaxel	1 (0.09)	0 (0.00)	0 (0.00)
Fluorouracil,Cisplatin,Trastuzumab	11 (1.00)	5 (1.00)	0 (0.00)
Fluorouracil,Clinical Study Drug,Leucovorin	1 (0.09)	0 (0.00)	0 (0.00)
Fluorouracil,Clinical Study Drug,Leucovorin,Bevacizumab	1 (0.09)	0 (0.00)	0 (0.00)

Fluorouracil,Clinical Study Drug,Leucovorin,Oxaliplatin	2 (0.18)	0 (0.00)	0 (0.00)
Fluorouracil,Clinical Study Drug,Oxaliplatin	0 (0.00)	1 (0.20)	0 (0.00)
Fluorouracil,Docetaxel,Leucovorin,Oxaliplatin	6 (0.54)	1 (0.20)	0 (0.00)
Fluorouracil,Docetaxel,Leucovorin,Oxaliplatin,Paclitaxel	1 (0.09)	0 (0.00)	0 (0.00)
Fluorouracil,Docetaxel,Oxaliplatin	7 (0.63)	2 (0.40)	1 (0.43)
Fluorouracil,Docetaxel,Oxaliplatin,Trastuzumab	1 (0.09)	0 (0.00)	0 (0.00)
Fluorouracil,Doxorubicin,Leucovorin	0 (0.00)	0 (0.00)	0 (0.00)
Fluorouracil,Epirubicin,Leucovorin	0 (0.00)	1 (0.20)	0 (0.00)
Fluorouracil,Epirubicin,Oxaliplatin	11 (1.00)	2 (0.40)	0 (0.00)
Fluorouracil,Etoposide,Leucovorin	0 (0.00)	1 (0.20)	0 (0.00)
Fluorouracil,Irinotecan,Leucovorin,Paclitaxel,Ramucirumab	0 (0.00)	1 (0.20)	0 (0.00)
Fluorouracil,Lapatinib,Leucovorin,Oxaliplatin	0 (0.00)	0 (0.00)	1 (0.43)
Fluorouracil,Leucovorin	8 (0.73)	0 (0.00)	0 (0.00)
Fluorouracil,Leucovorin,Leuprolide,Oxaliplatin	1 (0.09)	0 (0.00)	0 (0.00)
Fluorouracil,Leucovorin,Oxaliplatin,Paclitaxel	0 (0.00)	1 (0.20)	0 (0.00)
Fluorouracil,Leucovorin,Oxaliplatin,Paclitaxel,Trastuzumab	0 (0.00)	1 (0.20)	0 (0.00)
Fluorouracil,Leucovorin,Oxaliplatin,Triptorelin	1 (0.09)	0 (0.00)	0 (0.00)
Fluorouracil,Leucovorin,Ramucirumab	0 (0.00)	0 (0.00)	0 (0.00)
Fluorouracil,Leucovorin,Trastuzumab	2 (0.18)	0 (0.00)	2 (0.86)
Fluorouracil,Paclitaxel,Trastuzumab	0 (0.00)	1 (0.20)	0 (0.00)
Gemcitabine	0 (0.00)	1 (0.20)	1 (0.43)
Gemcitabine,Oxaliplatin	0 (0.00)	0 (0.00)	1 (0.43)
Gemcitabine,Paclitaxel Protein-Bound	0 (0.00)	1 (0.20)	0 (0.00)
Hydroxyurea	1 (0.09)	0 (0.00)	0 (0.00)
Ibrutinib	0 (0.00)	1 (0.20)	0 (0.00)
Ipilimumab,Nivolumab	0 (0.00)	0 (0.00)	0 (0.00)
Irinotecan	2 (0.18)	6 (1.20)	11 (4.74)
Irinotecan,Cetuximab	0 (0.00)	0 (0.00)	1 (0.43)
Irinotecan,Oxaliplatin	0 (0.00)	2 (0.40)	0 (0.00)
Irinotecan,Paclitaxel	0 (0.00)	1 (0.20)	0 (0.00)
Irinotecan,Paclitaxel,Ramucirumab	0 (0.00)	1 (0.20)	0 (0.00)

Irinotecan,Pembrolizumab	0 (0.00)	0 (0.00)	0 (0.00)
Irinotecan,Ramucirumab	0 (0.00)	1 (0.20)	2 (0.86)
Irinotecan,Trastuzumab	0 (0.00)	1 (0.20)	1 (0.43)
Letrozole	1 (0.09)	0 (0.00)	0 (0.00)
Leucovorin	0 (0.00)	1 (0.20)	0 (0.00)
Nivolumab	0 (0.00)	6 (1.20)	5 (2.16)
Oxaliplatin	1 (0.09)	0 (0.00)	0 (0.00)
Oxaliplatin,Ramucirumab	0 (0.00)	1 (0.20)	0 (0.00)
Oxaliplatin,Trastuzumab	0 (0.00)	0 (0.00)	1 (0.43)
Paclitaxel	4 (0.36)	14 (2.79)	5 (2.16)
Paclitaxel Protein-Bound,Ramucirumab	1 (0.09)	0 (0.00)	1 (0.43)
Paclitaxel,Pembrolizumab,Ramucirumab	0 (0.00)	1 (0.20)	0 (0.00)
Paclitaxel,Ramucirumab	23 (2.09)	84 (16.73)	43 (18.53)
Paclitaxel,Ramucirumab,Trastuzumab	0 (0.00)	2 (0.40)	0 (0.00)
Paclitaxel,Trastuzumab	4 (0.36)	3 (0.60)	2 (0.86)
Pazopanib	1 (0.09)	0 (0.00)	0 (0.00)
Pembrolizumab	4 (0.36)	7 (1.39)	12 (5.17)
Pembrolizumab,Ramucirumab	1 (0.09)	0 (0.00)	0 (0.00)
Pembrolizumab,Ruxolitinib	0 (0.00)	0 (0.00)	0 (0.00)
Pemetrexed	0 (0.00)	0 (0.00)	0 (0.00)
Ramucirumab	8 (0.73)	23 (4.58)	11 (4.74)
Ramucirumab,Trastuzumab	0 (0.00)	1 (0.20)	1 (0.43)
Ruxolitinib	0 (0.00)	1 (0.20)	1 (0.43)
Temsirolimus	1 (0.09)	0 (0.00)	0 (0.00)
Trastuzumab	4 (0.36)	4 (0.80)	3 (1.29)
Vinorelbine	1 (0.09)	0 (0.00)	1 (0.43)

Supplemental Table 3. Regimens received by patients with esophageal tumors

Regimen , n(%)	Line 1 (N=1359)	Line 2 (N=552)	Line 3 (N=227)
Abiraterone	0 (0.00)	1 (0.18)	0 (0.00)
Ado-Trastuzumab Emtansine	0 (0.00)	0 (0.00)	2 (0.88)
Ado-Trastuzumab Emtansine,Paclitaxel,Ramucirumab	1 (0.07)	0 (0.00)	0 (0.00)
Amifostine,Carboplatin,Paclitaxel	1 (0.07)	0 (0.00)	0 (0.00)
Anastrozole	0 (0.00)	1 (0.18)	0 (0.00)
Bevacizumab	1 (0.07)	1 (0.18)	0 (0.00)
Bicalutamide	0 (0.00)	0 (0.00)	1 (0.44)
Bortezomib,Paclitaxel	1 (0.07)	0 (0.00)	0 (0.00)
CAPEOX	22 (1.62)	12 (2.17)	5 (2.20)
CAPEOX,Trastuzumab	5 (0.37)	2 (0.36)	1 (0.44)
Capecitabine	36 (2.65)	30 (5.43)	8 (3.52)
Capecitabine,Carboplatin	1 (0.07)	0 (0.00)	0 (0.00)
Capecitabine,Carboplatin,Docetaxel	2 (0.15)	0 (0.00)	0 (0.00)
Capecitabine,Carboplatin,Epirubicin,Oxaliplatin,Paclitaxel	1 (0.07)	0 (0.00)	0 (0.00)
Capecitabine,Carboplatin,Irinotecan	1 (0.07)	0 (0.00)	0 (0.00)
Capecitabine,Carboplatin,Oxaliplatin,Paclitaxel	0 (0.00)	1 (0.18)	0 (0.00)
Capecitabine,Carboplatin,Paclitaxel	4 (0.29)	0 (0.00)	0 (0.00)
Capecitabine,Carboplatin,Paclitaxel,Trastuzumab	2 (0.15)	0 (0.00)	0 (0.00)
Capecitabine,Carboplatin, Trastuzumab	1 (0.07)	0 (0.00)	1 (0.44)
Capecitabine,Cisplatin	2 (0.15)	3 (0.54)	0 (0.00)
Capecitabine,Cisplatin,Docetaxel	1 (0.07)	0 (0.00)	0 (0.00)
Capecitabine,Cisplatin,Epirubicin	5 (0.37)	3 (0.54)	0 (0.00)
Capecitabine,Cisplatin,Trastuzumab	4 (0.29)	1 (0.18)	0 (0.00)
Capecitabine,Clinical Study Drug	0 (0.00)	1 (0.18)	0 (0.00)
Capecitabine,Docetaxel	0 (0.00)	1 (0.18)	0 (0.00)
Capecitabine,Docetaxel,Leucovorin	0 (0.00)	0 (0.00)	1 (0.44)
Capecitabine,Docetaxel,Paclitaxel,Ramucirumab	0 (0.00)	1 (0.18)	0 (0.00)
Capecitabine,Epirubicin	0 (0.00)	1 (0.18)	0 (0.00)

Capecitabine,Epirubicin,Oxaliplatin	34 (2.50)	10 (1.81)	2 (0.88)
Capecitabine,Epirubicin,Oxaliplatin, Trastuzumab	2 (0.15)	0 (0.00)	1 (0.44)
Capecitabine,Irinotecan	0 (0.00)	2 (0.36)	5 (2.20)
Capecitabine,Irinotecan,Leucovorin,Trastuzumab	0 (0.00)	0 (0.00)	1 (0.44)
Capecitabine,Paclitaxel	0 (0.00)	1 (0.18)	0 (0.00)
Capecitabine,Trastuzumab	1 (0.07)	3 (0.54)	0 (0.00)
Carboplatin	9 (0.66)	3 (0.54)	2 (0.88)
Carboplatin,Clinical Study Drug,Paclitaxel	2 (0.15)	0 (0.00)	0 (0.00)
Carboplatin,Docetaxel	13 (0.96)	4 (0.72)	2 (0.88)
Carboplatin,Docetaxel,Paclitaxel	2 (0.15)	0 (0.00)	0 (0.00)
Carboplatin,Docetaxel,Paclitaxel,Trastuzumab	1 (0.07)	0 (0.00)	0 (0.00)
Carboplatin,Docetaxel,Trastuzumab	2 (0.15)	3 (0.54)	0 (0.00)
Carboplatin,Enzalutamide,Paclitaxel	1 (0.07)	0 (0.00)	0 (0.00)
Carboplatin,Etoposide	1 (0.07)	1 (0.18)	0 (0.00)
Carboplatin,Irinotecan	0 (0.00)	3 (0.54)	1 (0.44)
Carboplatin,Irinotecan,Paclitaxel	0 (0.00)	1 (0.18)	0 (0.00)
Carboplatin,Irinotecan,Trastuzumab	0 (0.00)	0 (0.00)	1 (0.44)
Carboplatin,Leuprolide,Paclitaxel	0 (0.00)	1 (0.18)	0 (0.00)
Carboplatin,Paclitaxel	57 (4.19)	20 (3.62)	6 (2.64)
Carboplatin,Paclitaxel Protein-Bound	3 (0.22)	0 (0.00)	0 (0.00)
Carboplatin,Paclitaxel Protein-Bound,Trastuzumab	1 (0.07)	0 (0.00)	0 (0.00)
Carboplatin,Paclitaxel,Bevacizumab	1 (0.07)	0 (0.00)	0 (0.00)
Carboplatin,Paclitaxel,Cetuximab	1 (0.07)	0 (0.00)	0 (0.00)
Carboplatin,Paclitaxel,Pembrolizumab	1 (0.07)	0 (0.00)	0 (0.00)
Carboplatin,Paclitaxel,Radiation	400 (29.43)	28 (5.07)	1 (0.44)
Carboplatin,Paclitaxel,Ramucirumab	1 (0.07)	1 (0.18)	2 (0.88)
Carboplatin,Paclitaxel,Trametinib,Cetuximab	0 (0.00)	1 (0.18)	0 (0.00)
Carboplatin,Paclitaxel,Trastuzumab	25 (1.84)	4 (0.72)	1 (0.44)
Carboplatin,Pemetrexed	0 (0.00)	1 (0.18)	0 (0.00)
Cetuximab	0 (0.00)	1 (0.18)	0 (0.00)
Cisplatin	4 (0.29)	0 (0.00)	0 (0.00)

Cisplatin,Docetaxel	4 (0.29)	1 (0.18)	0 (0.00)
Cisplatin,Docetaxel,Trastuzumab	0 (0.00)	3 (0.54)	0 (0.00)
Cisplatin,Epirubicin	2 (0.15)	0 (0.00)	0 (0.00)
Cisplatin,Gemcitabine	0 (0.00)	1 (0.18)	0 (0.00)
Cisplatin,Irinotecan	6 (0.44)	2 (0.36)	3 (1.32)
Cisplatin,Irinotecan,Trastuzumab	0 (0.00)	0 (0.00)	2 (0.88)
Cisplatin,Paclitaxel	0 (0.00)	2 (0.36)	0 (0.00)
Cisplatin,Paclitaxel,Pembrolizumab,Ramucirumab	1 (0.07)	0 (0.00)	0 (0.00)
Cisplatin,Trastuzumab	1 (0.07)	1 (0.18)	0 (0.00)
Clinical Study Drug	18 (1.32)	13 (2.36)	5 (2.20)
Clinical Study Drug,Paclitaxel Protein-Bound	1 (0.07)	0 (0.00)	0 (0.00)
Clinical Study Drug,Paclitaxel,Trastuzumab	0 (0.00)	0 (0.00)	1 (0.44)
Clinical Study Drug,Trastuzumab	1 (0.07)	0 (0.00)	0 (0.00)
Crizotinib	0 (0.00)	1 (0.18)	0 (0.00)
Cromolyn,Trastuzumab	0 (0.00)	0 (0.00)	1 (0.44)
Cyclophosphamide	1 (0.07)	0 (0.00)	0 (0.00)
Cyclophosphamide,Dactinomycin,Etoposide,Leucovorin,Methotrexate,Vincristine	0 (0.00)	1 (0.18)	0 (0.00)
Cyclophosphamide,Doxorubicin,Rituximab,Vincristine	0 (0.00)	1 (0.18)	0 (0.00)
Dasatinib	0 (0.00)	1 (0.18)	0 (0.00)
Decitabine	1 (0.07)	0 (0.00)	0 (0.00)
Docetaxel	8 (0.59)	5 (0.91)	4 (1.76)
Docetaxel,Gemcitabine	1 (0.07)	0 (0.00)	0 (0.00)
Docetaxel,Gemcitabine,Ramucirumab	0 (0.00)	1 (0.18)	0 (0.00)
Docetaxel,Irinotecan	2 (0.15)	2 (0.36)	1 (0.44)
Docetaxel,Leucovorin,Oxaliplatin	0 (0.00)	1 (0.18)	0 (0.00)
Docetaxel,Leuprolide	0 (0.00)	0 (0.00)	0 (0.00)
Docetaxel,Paclitaxel	1 (0.07)	0 (0.00)	0 (0.00)
Docetaxel,Pertuzumab,Trastuzumab	0 (0.00)	1 (0.18)	0 (0.00)
Docetaxel,Ramucirumab	4 (0.29)	1 (0.18)	2 (0.88)
Docetaxel,Trastuzumab	2 (0.15)	2 (0.36)	3 (1.32)

Doxorubicin	1 (0.07)	0 (0.00)	0 (0.00)
Doxorubicin,Pembrolizumab	0 (0.00)	1 (0.18)	0 (0.00)
Enzalutamide	0 (0.00)	1 (0.18)	0 (0.00)
Enzalutamide,Leuprolide	0 (0.00)	0 (0.00)	0 (0.00)
Epirubicin	0 (0.00)	1 (0.18)	0 (0.00)
Epirubicin,Etoposide	0 (0.00)	1 (0.18)	0 (0.00)
Epirubicin,Oxaliplatin	1 (0.07)	1 (0.18)	0 (0.00)
Epirubicin,Oxaliplatin,Trastuzumab	0 (0.00)	1 (0.18)	0 (0.00)
Erlotinib,Paclitaxel,Cetuximab	0 (0.00)	0 (0.00)	1 (0.44)
Everolimus	1 (0.07)	0 (0.00)	1 (0.44)
FOLFIRI	17 (1.25)	30 (5.43)	19 (8.37)
FOLFIRI,Ramucirumab	0 (0.00)	3 (0.54)	0 (0.00)
FOLFIRI,Trastuzumab	3 (0.22)	8 (1.45)	2 (0.88)
FOLFOX	264 (19.43)	84 (15.22)	19 (8.37)
FOLFOX,Bevacizumab	2 (0.15)	0 (0.00)	0 (0.00)
FOLFOX,Ramucirumab	0 (0.00)	2 (0.36)	0 (0.00)
FOLFOX,Trastuzumab	52 (3.83)	25 (4.53)	1 (0.44)
FOLFOXIRI	1 (0.07)	2 (0.36)	1 (0.44)
FOLFOXIRI, Ramucirumab	1 (0.07)	0 (0.00)	0 (0.00)
Fluorouracil	16 (1.18)	2 (0.36)	0 (0.00)
Fluorouracil,Amifostine,Cisplatin,Trastuzumab	1 (0.07)	0 (0.00)	0 (0.00)
Fluorouracil,Carboplatin	7 (0.52)	0 (0.00)	2 (0.88)
Fluorouracil,Carboplatin,Docetaxel	14 (1.03)	1 (0.18)	1 (0.44)
Fluorouracil,Carboplatin,Docetaxel,Trastuzumab	0 (0.00)	1 (0.18)	0 (0.00)
Fluorouracil,Carboplatin,Leucovorin,Oxaliplatin,Paclitaxel	3 (0.22)	1 (0.18)	0 (0.00)
Fluorouracil,Carboplatin,Oxaliplatin,Paclitaxel	1 (0.07)	0 (0.00)	0 (0.00)
Fluorouracil,Carboplatin,Paclitaxel	14 (1.03)	1 (0.18)	0 (0.00)
Fluorouracil,Carboplatin,Paclitaxel,Trastuzumab	2 (0.15)	0 (0.00)	0 (0.00)
Fluorouracil,Carboplatin,Trastuzumab	1 (0.07)	1 (0.18)	0 (0.00)
Fluorouracil,Cisplatin	52 (3.83)	6 (1.09)	1 (0.44)
Fluorouracil,Cisplatin,Cetuximab	1 (0.07)	0 (0.00)	0 (0.00)

Fluorouracil,Cisplatin,Docetaxel	32 (2.35)	2 (0.36)	2 (0.88)
Fluorouracil,Cisplatin,Docetaxel,Leucovorin	9 (0.66)	2 (0.36)	1 (0.44)
Fluorouracil,Cisplatin,Docetaxel,Leucovorin,Trastuzumab	3 (0.22)	0 (0.00)	0 (0.00)
Fluorouracil,Cisplatin,Docetaxel,Oxaliplatin	2 (0.15)	0 (0.00)	0 (0.00)
Fluorouracil,Cisplatin,Docetaxel,Trastuzumab	2 (0.15)	2 (0.36)	0 (0.00)
Fluorouracil,Cisplatin,Epirubicin	16 (1.18)	0 (0.00)	2 (0.88)
Fluorouracil,Cisplatin,Epirubicin,Leucovorin	0 (0.00)	0 (0.00)	1 (0.44)
Fluorouracil,Cisplatin,Irinotecan,Leucovorin	2 (0.15)	0 (0.00)	0 (0.00)
Fluorouracil,Cisplatin,Leucovorin,Oxaliplatin	0 (0.00)	1 (0.18)	0 (0.00)
Fluorouracil,Cisplatin,Leucovorin,Trastuzumab	3 (0.22)	2 (0.36)	0 (0.00)
Fluorouracil,Cisplatin,Methotrexate, Trastuzumab	0 (0.00)	1 (0.18)	0 (0.00)
Fluorouracil,Cisplatin,Ramucirumab	1 (0.07)	0 (0.00)	0 (0.00)
Fluorouracil,Cisplatin,Trastuzumab	11 (0.81)	7 (1.27)	1 (0.44)
Fluorouracil,Clinical Study Drug,Leucovorin	1 (0.07)	1 (0.18)	0 (0.00)
Fluorouracil,Docetaxel	2 (0.15)	0 (0.00)	0 (0.00)
Fluorouracil,Docetaxel,Leucovorin,Oxaliplatin	4 (0.29)	1 (0.18)	0 (0.00)
Fluorouracil,Docetaxel,Leucovorin,Oxaliplatin, Trastuzumab	0 (0.00)	1 (0.18)	0 (0.00)
Fluorouracil,Docetaxel,Oxaliplatin	14 (1.03)	4 (0.72)	1 (0.44)
Fluorouracil,Docetaxel,Oxaliplatin,Paclitaxel	0 (0.00)	1 (0.18)	0 (0.00)
Fluorouracil,Docetaxel,Oxaliplatin, Trastuzumab	5 (0.37)	0 (0.00)	1 (0.44)
Fluorouracil,Epirubicin,Leucovorin,Oxaliplatin	1 (0.07)	0 (0.00)	0 (0.00)
Fluorouracil,Epirubicin,Oxaliplatin	3 (0.22)	0 (0.00)	1 (0.44)
Fluorouracil,Epirubicin,Oxaliplatin, Trastuzumab	1 (0.07)	1 (0.18)	0 (0.00)
Fluorouracil,Irinotecan,Leucovorin,Pembrolizumab	0 (0.00)	0 (0.00)	2 (0.88)
Fluorouracil,Lapatinib,Oxaliplatin,Trastuzumab	1 (0.07)	0 (0.00)	0 (0.00)
Fluorouracil,Leucovorin	8 (0.59)	5 (0.91)	1 (0.44)
Fluorouracil,Leucovorin,Methotrexate,Oxaliplatin	1 (0.07)	0 (0.00)	0 (0.00)
Fluorouracil,Leucovorin,Mitomycin,Oxaliplatin	0 (0.00)	1 (0.18)	0 (0.00)
Fluorouracil,Leucovorin,Olaparib,Oxaliplatin	0 (0.00)	1 (0.18)	0 (0.00)
Fluorouracil,Leucovorin,Oxaliplatin,Paclitaxel,Ramucirumab	0 (0.00)	1 (0.18)	0 (0.00)
Fluorouracil,Leucovorin,Oxaliplatin,Pembrolizumab	0 (0.00)	1 (0.18)	0 (0.00)

Fluorouracil,Leucovorin,Pembrolizumab	0 (0.00)	0 (0.00)	1 (0.44)
Fluorouracil,Leucovorin,Trastuzumab	1 (0.07)	3 (0.54)	0 (0.00)
Fluorouracil,Mitomycin	1 (0.07)	0 (0.00)	0 (0.00)
Fluorouracil,Oxaliplatin,Paclitaxel	1 (0.07)	0 (0.00)	0 (0.00)
Fluorouracil,Oxaliplatin,Paclitaxel,Trastuzumab	1 (0.07)	0 (0.00)	0 (0.00)
Fluorouracil,Oxaliplatin,Pembrolizumab	0 (0.00)	0 (0.00)	0 (0.00)
Fluorouracil,Paclitaxel Protein-Bound	0 (0.00)	1 (0.18)	0 (0.00)
Fluorouracil,Trastuzumab	1 (0.07)	0 (0.00)	0 (0.00)
Gemcitabine	1 (0.07)	0 (0.00)	0 (0.00)
Gemcitabine,Paclitaxel Protein-Bound	1 (0.07)	0 (0.00)	0 (0.00)
Imatinib,Topotecan	0 (0.00)	0 (0.00)	1 (0.44)
Interferon Alfa-2B,Trastuzumab	0 (0.00)	1 (0.18)	0 (0.00)
Irinotecan	6 (0.44)	11 (1.99)	9 (3.96)
Irinotecan,Ramucirumab	0 (0.00)	1 (0.18)	3 (1.32)
Irinotecan,Trastuzumab	0 (0.00)	4 (0.72)	1 (0.44)
Lenvatinib	1 (0.07)	0 (0.00)	0 (0.00)
Letrozole	1 (0.07)	0 (0.00)	0 (0.00)
Leucovorin,Oxaliplatin	0 (0.00)	1 (0.18)	0 (0.00)
Leuprolide	2 (0.15)	1 (0.18)	1 (0.44)
Nivolumab	2 (0.15)	8 (1.45)	10 (4.41)
Nivolumab,Ramucirumab	0 (0.00)	0 (0.00)	0 (0.00)
Nivolumab,Trastuzumab	0 (0.00)	0 (0.00)	1 (0.44)
Oxaliplatin	2 (0.15)	1 (0.18)	0 (0.00)
Oxaliplatin,Paclitaxel	1 (0.07)	0 (0.00)	0 (0.00)
Oxaliplatin,Ramucirumab	0 (0.00)	0 (0.00)	1 (0.44)
Oxaliplatin,Trastuzumab	0 (0.00)	1 (0.18)	0 (0.00)
Paclitaxel	10 (0.74)	9 (1.63)	3 (1.32)
Paclitaxel Protein-Bound	0 (0.00)	1 (0.18)	0 (0.00)
Paclitaxel Protein-Bound,Ramucirumab	0 (0.00)	0 (0.00)	0 (0.00)
Paclitaxel,Pembrolizumab,Ramucirumab	0 (0.00)	0 (0.00)	1 (0.44)
Paclitaxel,Ramucirumab	20 (1.47)	76 (13.77)	36 (15.86)

Paclitaxel,Ramucirumab,Trastuzumab	0 (0.00)	2 (0.36)	2 (0.88)
Paclitaxel,Trastuzumab	1 (0.07)	4 (0.72)	0 (0.00)
Pazopanib	0 (0.00)	0 (0.00)	1 (0.44)
Pembrolizumab	3 (0.22)	11 (1.99)	14 (6.17)
Ramucirumab	6 (0.44)	14 (2.54)	12 (5.29)
Ramucirumab,Trastuzumab	1 (0.07)	1 (0.18)	1 (0.44)
Rituximab	1 (0.07)	0 (0.00)	0 (0.00)
Samarium Sm 153 Lexidronam	0 (0.00)	0 (0.00)	0 (0.00)
Sipuleucel-T	1 (0.07)	0 (0.00)	0 (0.00)
Sorafenib	0 (0.00)	1 (0.18)	1 (0.44)
Trametinib	0 (0.00)	0 (0.00)	0 (0.00)
Trastuzumab	9 (0.66)	4 (0.72)	1 (0.44)
Vinorelbine	1 (0.07)	1 (0.18)	0 (0.00)
